# An integrated climate-structured personal and ambient air pollution monitoring framework embedded within a pregnancy and postpartum cohort in Sub-Saharan Africa

**DOI:** 10.3389/fgwh.2026.1849628

**Published:** 2026-10-05

**Authors:** Liberty Makacha, Jovito Nune, P. Tatenda Makanga, Cathryn Tonne, Marie-Laure Volvert, Hawanatu Jah, Yahaya Idris, Esperança Sevene, Moses Mukhanya, Angela Koech, Onesmus Wanje, Anifa Valá, Hannah Blencowe, Umberto d’Alessandro, Marleen Temmerman, Anna Roca, Jeffrey N. Bone, Laura A. Magee, Peter von Dadelszen, Ben Barratt, the PRECISE and PRECISE-DYAD Networks

**Affiliations:** 1 Department of Women and Children’s Health, King’s College London, London, United Kingdom; 2 Place Alert Labs, Midlands State University, Gweru, Zimbabwe; 3 Centre for Sexual Health and HIV AIDS Research Zimbabwe (CeSHHAR), Harare, Zimbabwe; 4 ISGlobal, Barcelona Institute for Global Health, Universitat de Barcelona, Barcelona, Spain; 5 Department of International Public Health, Liverpool School of Tropical Medicine, Liverpool, United Kingdom; 6 MRC Unit The Gambia at the London School of Hygiene and Tropical Medicine, Banjul, Gambia; 7 Centro de Investigação em Saúde de Manhiça, Manhiça, Mozambique; 8 Faculty of Medicine, Eduardo Mondlane University, Maputo, Mozambique; 9 Centre of Excellence for Women and Child Health, Aga Khan University, Nairobi, East Africa, Kenya; 10 London School of Hygiene and Tropical Medicine, London, United Kingdom; 11 ICREA, Barcelona, Spain; 12 British Columbia Children’s Hospital Research Institute, Vancouver, BC, Canada; 13 School of Public Health, Imperial College London, London, United Kingdom

**Keywords:** air pollution exposure assessment, climate variability, maternal health, microenvironment, personal exposure monitoring, PM₂.₅, Sub-Saharan Africa, wearable sensors

## Abstract

**Background:**

In sub-Saharan Africa, climatic variability intersects with structural inequities—biomass reliance, informal livelihoods, rapid urbanisation, and sparse regulatory monitoring—to generate highly heterogeneous air pollution exposures during pregnancy. Maternal exposure assessment in the region remains dominated by coarse ambient estimates, limiting understanding of how climate and microenvironment jointly shape actual personal exposure through their influence on pollutant generation, dispersion, and proximity to emission sources.

**Objective:**

To develop and implement an adaptable, climate-structured personal and ambient PM₂.₅ monitoring framework embedded within the PRECISE pregnancy cohort across The Gambia, Kenya, and Mozambique.

**Methods:**

A purposively sampled subset of 343 pregnant or postpartum women (The Gambia *n* = 160; Kenya *n* = 105; Mozambique *n* = 78) completed two 5-day deployments (with usable data) of wearable multi-parameter monitors in opposing dry and wet seasons. Devices recorded minute-resolved PM₂.₅, temperature, humidity, and mobility (GPS/accelerometry). Fixed-site monitors were installed at six health facilities to characterise background exposure and seasonal dynamics. Calibration combined repeated pre-deployment co-location with reference instrumentation and in-country inter-sensor harmonisation using Deming regression. Quality assurance incorporated a ≥75% ambient air pollution data completeness threshold, encrypted transfer, geolocation validation, and cross-site reproducibility checks. Meteorological data (NASA POWER/MERRA-2) were integrated to contextualise climatic drivers.

**Results:**

The protocol generated 3,190 person-days of personal exposure data together with 328 unique days of continuous ambient data. Ambient datasets achieved high completeness in The Gambia and Kenya (>85% data capture); Mozambique ambient capture was lower due to site-level connectivity constraints. Ambient monitoring periods were included only when ≥75% of expected observations were available; periods not meeting this threshold were excluded from primary analyses. Inter-sensor agreement was robust (R^2^ > 0.72 pre-deployment; >0.75 in-country). Ambient monitoring characterised seasonal background variability and enabled evaluation of ambient–personal concordance under contrasting climatic regimes. Integration of personal and ambient datasets supported microenvironmental partitioning and climate-contextual interpretation of exposure patterns.

**Conclusion:**

This integrated monitoring framework extends the operational and methodological capacity for high-resolution exposure assessment through the generation of high-resolution, climate-structured exposure data within pregnancy cohorts in under-monitored African settings, advancing equitable exposure science and strengthening climate-informed maternal and newborn health research.

**Significance statement:**

This study establishes an adaptable, climate-structured exposure measurement architecture that reduces exposure misclassification and enables more precise exposure–response inference in maternal health research conducted in under-monitored environments

## Introduction

Air pollution exposure assessment remains a major methodological challenge, particularly in regions with limited regulatory monitoring infrastructure. In sub-Saharan Africa (SSA), climatic variability interacts with structural and microenvironmental conditions to influence pollutant generation, atmospheric dispersion, and individual proximity to emission sources, thereby producing highly heterogeneous personal exposure profiles (1–4). Seasonal oscillations in precipitation, temperature, wind fields, and atmospheric mixing depth influence pollutant formation, transport, and persistence across rural and peri-urban environments, producing substantial spatial and temporal variability in ambient concentrations (5–8). However, routine monitoring infrastructure in many SSA settings remains insufficient to characterise these dynamics at adequate spatial resolution (9).

Fine particulate matter (PM₂.₅) is of particular concern given its small aerodynamic diameter and capacity to penetrate the distal lung and translocate into systemic circulation, contributing to oxidative stress and inflammatory responses (10–14). Although epidemiological evidence linking PM₂.₅ exposure to adverse health outcomes has expanded substantially in high-income settings, exposure assessment across SSA remains constrained by limited ground-based monitoring and reliance on modelled or satellite-derived ambient estimates (4, 15). While these approaches provide valuable regional estimates, they often inadequately capture the microenvironmental and behavioural determinants of lived exposure in contexts where indoor biomass combustion, peri-domestic cooking, unpaved roads, transport corridors, and seasonal dust mobilisation contribute materially to particulate concentrations (16).

This limitation is both methodological and structural. Ambient concentrations measured at fixed sites may poorly represent individual exposure where mobility patterns, housing ventilation characteristics, and occupational activities influence proximity to emission sources (17–19). Moreover, exposure distributions in such environments are frequently right-skewed, characterised by episodic high-intensity events superimposed on moderate background concentrations (20, 21). Without temporally resolved, person-centred monitoring, exposure–response analyses risk misclassification and attenuated effect estimates (22, 23).

Structural determinants further complicate exposure assessment. Wealth gradients, occupational roles, rural–urban residence, and household energy access may modify both baseline concentrations and seasonal amplification (24). Climatic variability does not operate uniformly across social strata; rather, it interacts with housing typology, mobility routines, and local emission sources to produce differential exposure patterns across communities (25, 26).

Recent advances in wearable sensing technologies offer opportunities to address these limitations by enabling high-resolution personal monitoring integrated with geolocation and environmental context data (27, 28). Implementing such systems in climatically diverse and resource-constrained environments presents operational and methodological challenges. Optical particle counters require careful calibration and harmonisation to address inter-sensor variability, drift, and humidity sensitivity (29). Field deployment must also ensure device durability, secure data handling, and reproducible processing pipelines capable of supporting multi-site studies (30). Monitoring frameworks must therefore be scalable—capable of generating intensive exposure measurements in subsets while informing exposure modelling for larger study populations (31). Beyond technical performance, successful deployment of wearable monitoring technologies requires social acceptability and community trust. In many low-resource settings, unfamiliar environmental monitoring devices may raise concerns regarding privacy, surveillance, or disruption of routine activities. Consequently, community engagement, participant sensitisation, and culturally responsive device design are increasingly recognised as integral components of successful exposure assessment frameworks rather than ancillary implementation activities. Embedding these processes within protocol development may improve participant adherence, reduce refusal rates, and enhance data completeness in intensive monitoring studies.

To address these methodological constraints, we developed and implemented an integrated personal and ambient PM₂.₅ monitoring protocol for PRECISE Air Quality research across The Gambia, Kenya, and Mozambique (32–34), following up women who had participated in the PRECISE study of unselected pregnant women recruited at the time of booking for antenatal care (35, 59). The framework combines minute-resolution wearable monitoring with concurrent facility-based ambient measurements, repeated seasonal deployments, structured calibration procedures, geolocation-informed microenvironment classification, and harmonised data governance protocols within a pregnancy cohort.

Specifically, the objectives of this manuscript are to: (i) describe the conceptual design of the PRECISE Air Quality protocol; (ii) document the calibration, harmonisation, and data governance procedures implemented across study sites; (iii) evaluate protocol implementation and data quality; and (iv) provide a reproducible framework for climate-structured exposure assessment in under-monitored settings. This study presents an adaptable, climate-structured exposure measurement architecture, evaluated through multi-country implementation, rather than a descriptive exposure analysis. Rather than presenting exposure distributions or health associations, our aim is to detail the operational and analytical architecture necessary to generate reliable, climate-contextualised exposure data for maternal and newborn health research. Site-specific exposure characterisation and exposure–response analyses are reported separately in companion analytical manuscripts.

## The PRECISE air quality study conceptual exposure framework

The integrated exposure assessment framework, linking personal monitoring, microenvironment classification, and ambient context, is illustrated in Figure 1.

**Figure 1:**
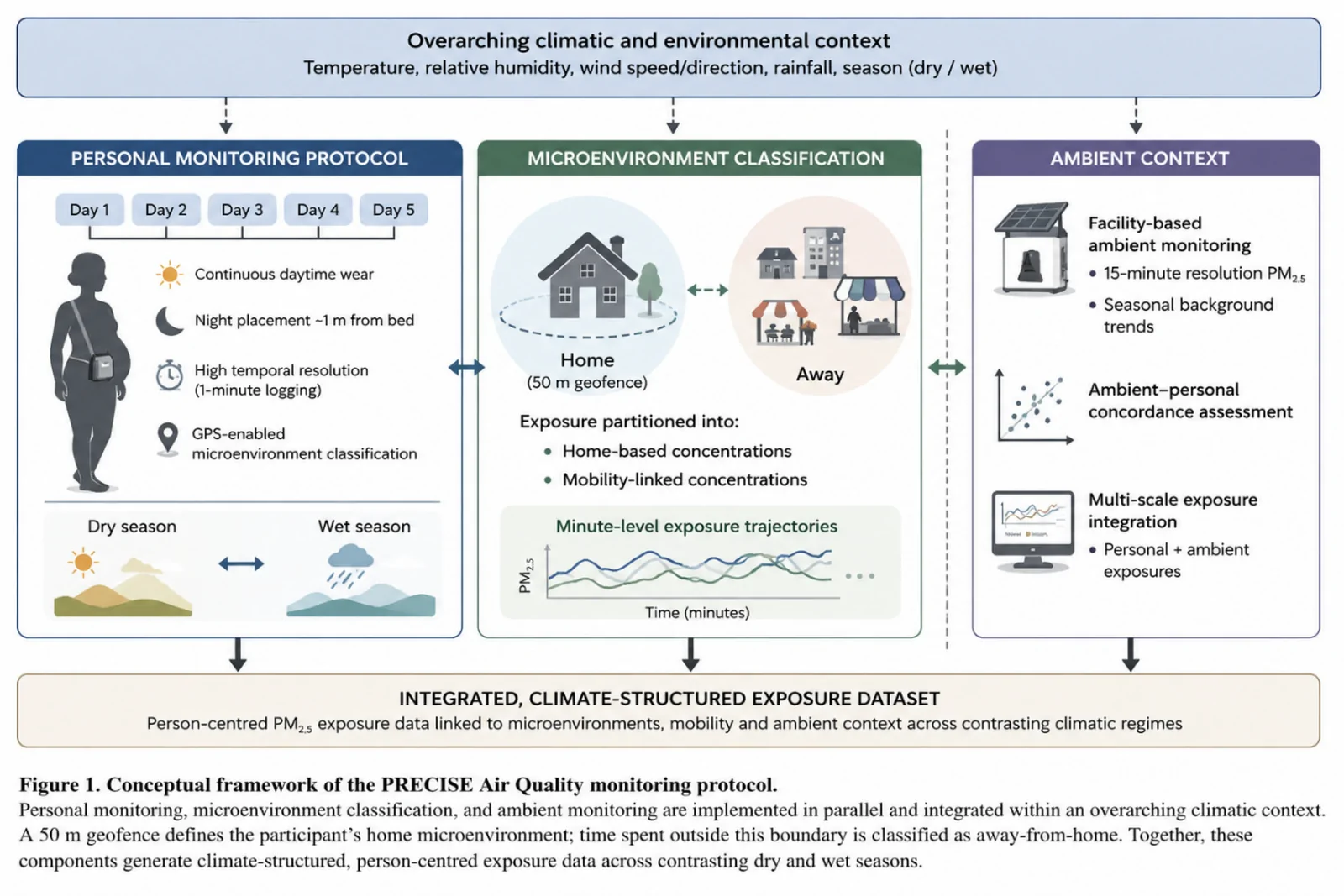
Conceptual exposure framework.

Conceptual framework for integrated exposure assessment: Personal PM₂.₅ monitoring was conducted at minute-level resolution over repeated seasonal deployments, with GPS-enabled classification of microenvironments into home and mobility-linked domains. Ambient monitoring provided contextual background concentrations at 15-minute resolution. These components were integrated to characterise individual exposure trajectories across contrasting climatic regimes. A 50 m geofence represents the predefined spatial buffer used to classify home-based exposure and was selected to accommodate expected GPS positional uncertainty under field conditions in the study settings.

Although the methodological framework described here is broadly applicable to environmental epidemiology, pregnancy and the postpartum period provide a particularly relevant context for intensive exposure assessment. Women may experience elevated exposure to household and community pollution sources through cooking, caregiving, and livelihood activities, while physiological adaptations during and after pregnancy may modify susceptibility to environmental stressors. Embedding exposure monitoring within maternal cohorts therefore provides an opportunity to improve exposure characterisation during a critical life stage.

## Methods

The PRECISE Air Quality study was implemented as a multi-country repeated-measures exposure monitoring study embedded within the PRECISE cohort and its extensions in The Gambia, Kenya, and Mozambique. The framework integrated repeated personal PM₂.₅ monitoring, concurrent facility-based ambient monitoring, sensor calibration and harmonisation, geolocation-based microenvironment classification, and meteorological contextualisation. The overall study design and cohort structure are summarised in Figure 2.

**Figure 2:**
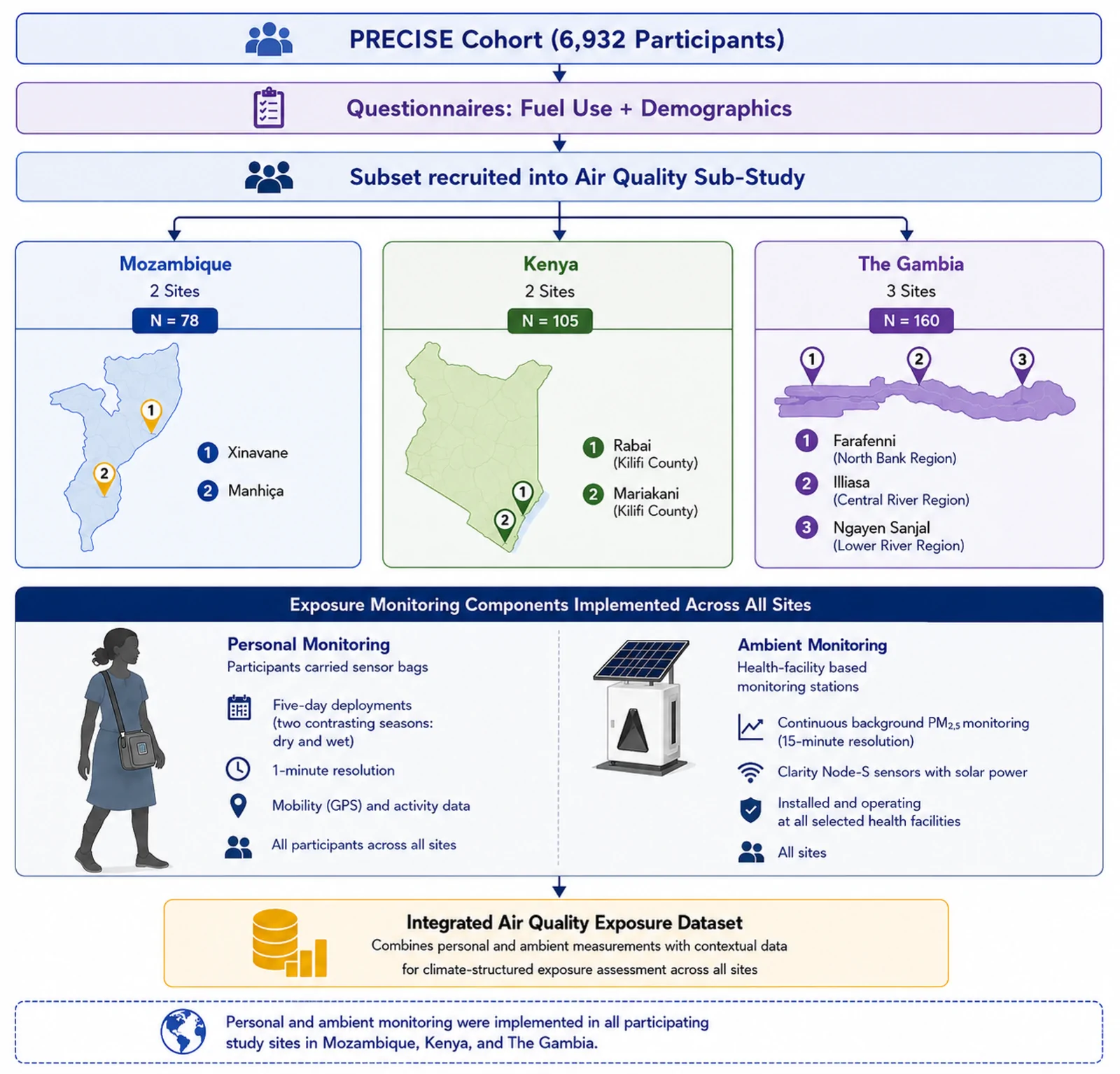
Study design and cohort structure of the PRECISE air quality study.

Study Design and Framework

The PRECISE Air Quality study was implemented as a multi-country repeated-measures exposure monitoring study embedded within the PRECISE cohort and its extensions in The Gambia, Kenya, and Mozambique. The study was designed to integrate person-level and contextual environmental measurements within a common exposure assessment framework. This comprised repeated personal PM₂.₅ monitoring, concurrent fixed-site ambient monitoring, geolocation- and activity-informed microenvironment classification, meteorological contextualisation, and standardised calibration, harmonisation, and data-processing procedures across study settings.

Personal and ambient measurements were collected as complementary components of the framework. Personal monitoring was used to capture individual exposure arising from participants’ movements and activities across different microenvironments, while fixed-site monitoring characterised background PM₂.₅ concentrations within participating health-facility catchment areas. These data streams were subsequently aligned temporally and integrated with geolocation, activity, meteorological, and participant-level contextual information to support climate-structured exposure assessment.

The overall study architecture, including the relationship between the parent cohort, air-quality sub-study, participating study settings, personal monitoring, ambient monitoring, and integrated exposure dataset, is summarised in Figure 2.

The air-quality sub-study was embedded within the wider PRECISE cohort and implemented across participating sites in Mozambique, Kenya, and The Gambia. The framework combined repeated personal exposure monitoring with health-facility-based ambient PM₂.₅ monitoring and contextual participant data. Personal, ambient, geolocation, activity, and contextual measurements were subsequently integrated within a harmonised exposure dataset for climate-structured exposure assessment.

## Study settings

Field implementation was conducted between March 2022 and January 2023 across seven health-facility catchment areas in three countries. In Mozambique, activities were centred at Manhiça District Hospital and Xinavane Rural Hospital; in Kenya, at Mariakani Sub-County Hospital and Rabai Health Centre; and in The Gambia, through Farafenni Hospital and associated rural facilities serving Illiasa and Ngayen Sanjal. The sites were purposively selected to represent variation in climatic conditions, settlement characteristics, emission environments, and structural determinants relevant to air pollution exposure.

## Participant sampling

A total of 343 participants were recruited across the three countries (The Gambia *n* = 160; Kenya *n* = 105; Mozambique *n* = 78). Participants were purposively sampled to capture heterogeneity across determinants influencing exposure, including household fuel use, ventilation characteristics, occupational activity, rural–urban residence, and socioeconomic status.

## Seasonal monitoring design

Participants were scheduled to complete two personal exposure monitoring deployments, each lasting five consecutive days (120 h), with one deployment undertaken during the dry season and the second during the wet season. This repeated seasonal design was intended to capture within-participant variation in exposure under contrasting climatic conditions while maintaining a standardised monitoring duration across study settings.

During each deployment, participants were asked to carry the wearable monitor throughout their normal waking activities. During periods of rest or sleep, the device was positioned close to the participant to approximate breathing-zone exposure while minimising disruption to usual activities. Monitoring continued throughout the five-day deployment, with PM₂.₅ and associated environmental and activity measurements recorded at one-minute resolution.

The timing of deployments was organised by the respective country field teams to coincide with the contrasting dry and wet seasonal periods within each study setting. Where participants completed both deployments, the resulting measurements provided repeated observations under different seasonal conditions. Eligibility of individual deployments for analysis was subsequently determined using the predefined data-completeness criteria described under Data Processing.

## Personal exposure monitoring

The field-deployed monitoring equipment, including wearable sensor configuration and supporting hardware, is shown in Figure 3.

**Figure 3:**
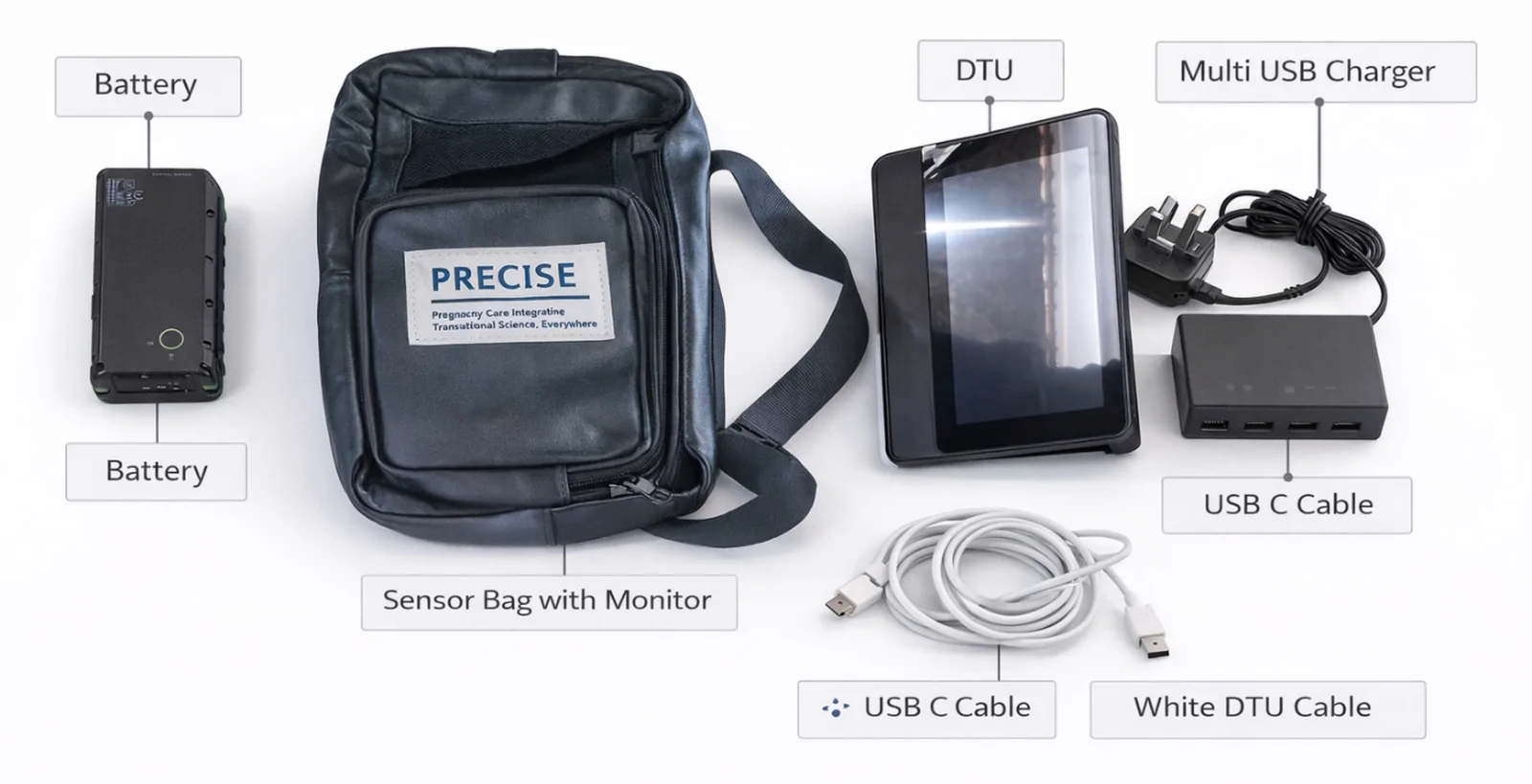
PRECISE air quality personal monitoring equipment.

Figure 3 above details the field-deployed equipment used for personal exposure monitoring. The wearable PRECISE sensor bag housed the multi-parameter monitoring unit, including the Sensirion SPS30 optical particle counter and associated environmental, GPS and motion sensors, together with a portable battery supply. Supporting equipment included a data transfer unit (DTU), multi-USB charger, USB charging cables and associated power connections. The integrated system enabled continuous minute-resolution personal PM₂.₅ and contextual environmental monitoring during five-day field deployments, enabling characterisation of fine-scale temporal and spatial variability in personal exposure.

## Community engagement and participant sensitisation

Community engagement was integrated throughout protocol implementation to support participant acceptability, adherence, and data completeness. Prior to deployment, engagement activities were conducted with community leaders, health facility staff, and prospective participants to explain study objectives, monitoring procedures, and data governance safeguards. Emphasis was placed on clarifying the function of wearable monitoring devices, addressing concerns regarding potential audio or visual recording, and explaining the purpose of GPS-enabled monitoring and geolocation data collection.

Participants received demonstrations of monitoring equipment and opportunities to discuss study procedures before enrolment. Throughout data collection, fieldworkers maintained regular contact with participants through mid-deployment visits, troubleshooting support, and ongoing communication with community stakeholders. These activities facilitated understanding of study procedures, reinforced confidence in the monitoring process, and supported adherence to repeated 5-day monitoring deployments across contrasting seasonal conditions.

Community engagement was therefore implemented not as an ancillary activity, but as a core component of the exposure assessment framework, complementing technical quality assurance procedures and contributing to successful protocol implementation across diverse sociocultural settings.

Complementary qualitative research was undertaken within the Kenya study sites to examine participant and fieldworker experiences of personal exposure monitoring, including device acceptability, perceptions of monitoring, and implementation within everyday activities; these findings will be reported separately.

## Ambient monitoring

Ambient PM₂.₅ concentrations were measured using fixed-site monitors installed at two health facilities per country. Monitors were positioned to represent background concentrations within facility catchment areas and recorded measurements at 15-minute resolution. In Mozambique, supplementary reference measurements from a Beta Attenuation Monitor were available for contextual comparison. Together, these measurements supported characterisation of background concentrations, evaluation of ambient–personal relationships, and interpretation of seasonal dispersion patterns.

## Calibration and harmonisation

The calibration and inter-sensor harmonisation workflow, including laboratory co-location and field validation procedures, is presented in Figure 4.

**Figure 4:**
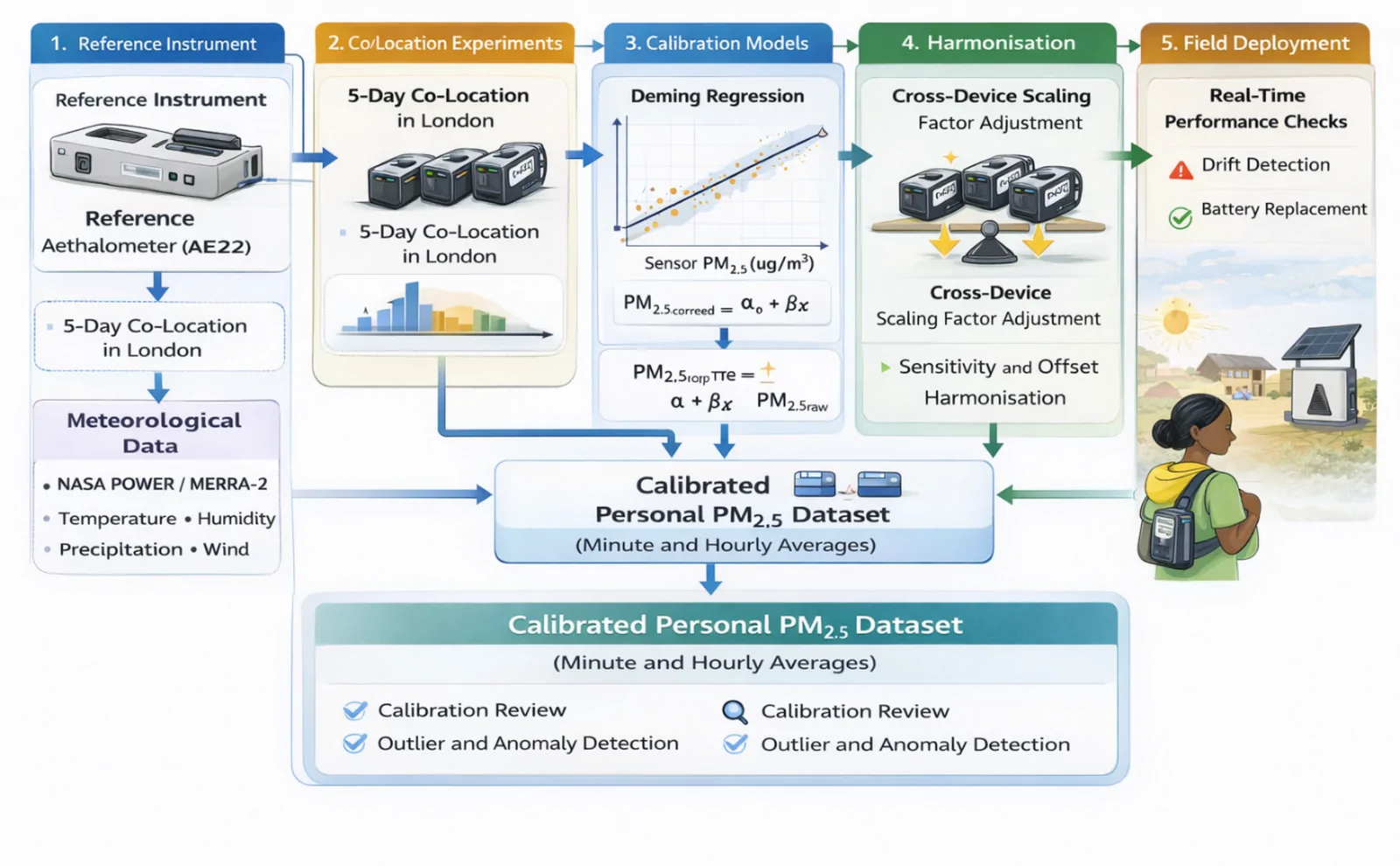
PRECISE air quality calibration and inter-sensor harmonisation framework.

Schematic representation of the sequential procedures used to improve comparability of personal PM₂.₅ measurements across devices and study settings. Wearable monitors underwent pre-deployment laboratory co-location in London, followed by calibration modelling using Deming regression and cross-device scaling. Before field deployment, monitors assigned to each study site underwent five-day in-country co-location to assess inter-sensor agreement and identify sensor drift. Meteorological information was incorporated to contextualise environmental conditions, and calibrated outputs were subjected to quality-control and field-performance checks before generation of the harmonised personal PM₂.₅ dataset. AE22, Aethalometer model AE22; PM₂.₅, particulate matter with aerodynamic diameter ≤2.5 μm; MERRA-2, Modern-Era Retrospective Analysis for Research and Applications, Version 2.

Devices exhibiting evidence of excessive drift, unstable calibration coefficients, or persistent divergence from the group mean were withdrawn from deployment pending inspection and recalibration. Harmonisation extended beyond sensor calibration. All devices were synchronised to local time before deployment using a standardised configuration protocol, and downloaded datasets were subjected to automated checks for timestamp consistency, duplicate records, and temporal discontinuities. Raw data from all sites were processed through an identical analytical workflow implemented in R, ensuring consistent application of calibration coefficients, quality-control procedures, exposure metric derivation, and geolocation-based microenvironment classification across countries. Detailed calibration specifications are provided in Supplementary Methods S3.

## Data processing

To ensure transparency and reproducibility, the full data processing and exposure derivation pipeline is summarised in Figure 5.

**Figure 5:**
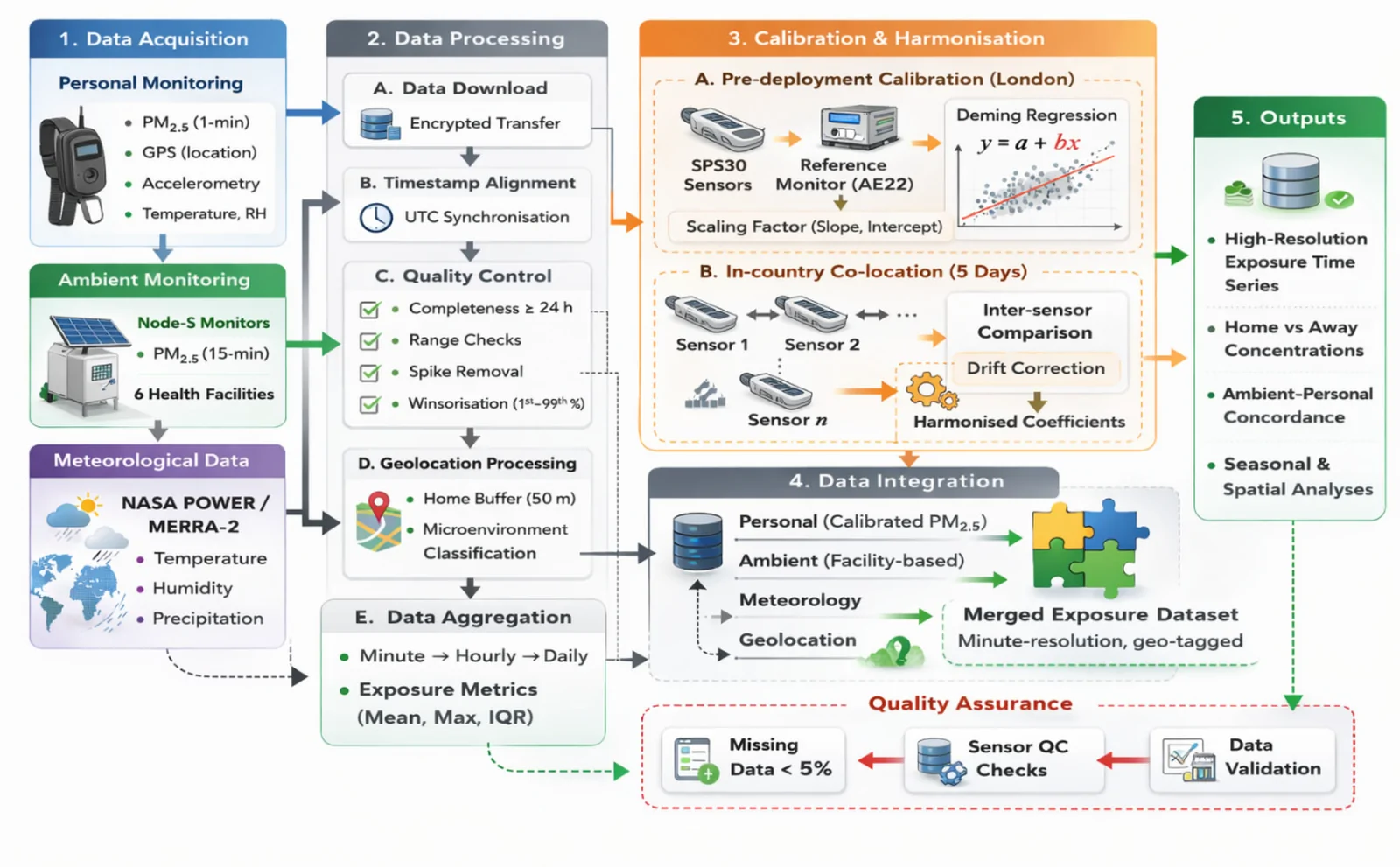
PRECISE air quality data pipeline.

Data processing workflow for personal and ambient PM₂.₅ measurements: Raw sensor data underwent quality control, time synchronisation, and calibration before aggregation to analytically relevant temporal scales. GPS data were used to classify microenvironments and partition exposure into home-based and mobility-linked components. Personal and ambient datasets were subsequently integrated to generate harmonised exposure metrics for epidemiological analysis.

Raw data from all three countries were processed using the same predefined workflow implemented in R. This workflow comprised timestamp standardisation and alignment; application of device-specific calibration coefficients; identification of duplicate, missing, or temporally discontinuous records; assessment of data completeness; treatment of extreme values; linkage of GPS and motion data; classification of home and mobility-linked microenvironments; temporal aggregation to minute, hourly, and daily exposure metrics; and integration of personal, ambient, and meteorological datasets. Applying the same processing rules and code across countries ensured that exposure metrics were derived consistently irrespective of study site. Devices were time-synchronised prior to deployment to ensure alignment across data streams.

A deployment was eligible for analysis when it contained at least 24 cumulative hours of valid PM₂.₅ measurements within the scheduled 120-hour deployment window; these observations were not required to constitute a single uninterrupted 24-hour period. This is consistent with recommendations from personal exposure assessment studies demonstrating that complete 24-hour periods provide stable estimates of daily exposure patterns (36). Extreme values were Winsorised at the 1st and 99th percentiles to reduce the influence of potential artefacts while retaining genuine high-exposure events, a robust statistical approach commonly used to minimise the impact of extreme observations without excluding them entirely (37).

Fixed-site ambient monitors operated continuously during the site-specific air-quality monitoring campaign, spanning the periods during which personal monitoring deployments were undertaken. Ambient PM₂.₅ concentrations were recorded at 15-minute intervals, yielding up to 96 observations per complete 24-hour day. For analyses requiring personal–ambient alignment, ambient observations corresponding temporally to the relevant personal monitoring periods were selected and synchronised by timestamp. Ambient monitoring periods were considered valid when at least 75% of the expected 15-minute observations were available for the defined analysis period. Periods falling below this threshold were excluded from analysis.

## Microenvironment classification

Geolocation data were used to classify exposure into domestic and mobility-linked environments. A 50 m radius around the participant's residence defined the home microenvironment. This threshold was selected *a priori* to accommodate expected GPS positional uncertainty under field conditions while remaining sufficiently sensitive to distinguish residential and mobility-linked environments, consistent with approaches used in previous personal exposure studies (38, 39).

Motion data supported contextual interpretation of exposure patterns and differentiation between stationary and active periods.

## Meteorological integration

Hourly meteorological variables were obtained from the NASA POWER platform. Variables included temperature, relative humidity, wind speed, and wind direction, enabling interpretation of seasonal variability and atmospheric dispersion. These data are derived from the MERRA-2 atmospheric reanalysis and were extracted at a spatial resolution of approximately 0.5° latitude×0.625° longitude and an hourly temporal resolution. Meteorological observations were linked to exposure measurements based on geographic location and timestamp to facilitate interpretation of seasonal and atmospheric dispersion patterns. Meteorological data were extracted for the coordinates corresponding to each study location.

## Protocol evaluation and statistical analysis framework

Analyses were structured around the four objectives of the manuscript and were designed to evaluate protocol implementation, measurement performance, cross-site harmonisation, and integration of personal and contextual exposure data. All analyses were conducted using R (version 4.4.0).

Protocol implementation and data completeness were evaluated using recruitment, refusal, deployment completion, monitoring duration, and valid-data capture metrics. Personal-monitoring completeness was assessed against the predefined minimum valid-monitoring criterion, while ambient completeness was calculated as the proportion of expected 15-minute observations available within each defined monitoring period.

Calibration and harmonisation performance were evaluated using Deming regression during laboratory and in-country co-location, with scaling factors, intercepts, coefficients of determination (R^2^), and evidence of sensor drift used to assess inter-device comparability.

## Personal–ambient measurement concordance

Because PM₂.₅ concentrations were right-skewed, concordance was assessed using ordinary least-squares regression after log₁₀ transformation of both personal and ambient hourly median concentrations:

$$\mathrm{lo}g_{10}\left(P_{it}\right)=\alpha +\beta \mathrm{lo}g_{10}\left(A_{t}\right)+\epsilon_{it}$$

where $P_{it}$ represents the hourly median personal PM₂.₅ concentration for participant *i* at time and represents the corresponding hourly median ambient PM₂.₅ concentration. Regression slopes and adjusted coefficients of determination (adjusted R^2^) were used to characterise the strength and direction of ambient–personal concordance. Analyses were stratified by study site and season to assess variation in concordance across geographic and climatic contexts

Microenvironment classification performance was evaluated as the proportion of deployments for which geolocation data permitted classification into home and mobility-linked environments using the predefined 50 m residential geofence. Exposure summaries were described using medians and interquartile ranges because PM₂.₅ distributions were right-skewed.

Protocol acceptability and field feasibility were evaluated descriptively using refusal rates, deployment completion, device-related data loss, and structured implementation observations recorded by field teams. Data-governance performance was assessed from successful encrypted transfer, linkage, storage, and documented data-loss or security events.

Detailed exposure distributions and exposure–response analyses were outside the scope of the present protocol evaluation and are reported in companion analytical manuscripts. Detailed operational procedures, calibration specifications, and quality assurance protocols are provided in Supplementary Methods S1–S6.

## Results

### Study implementation and data capture

Implementation outcomes are presented in relation to the protocol components described in the Methods. A total of 343 participants were enrolled across three countries (The Gambia *n* = 160; Kenya *n* = 105; Mozambique *n* = 78), each completing up to two five-day monitoring deployments in climatically contrasting seasons. Only five individuals declined participation across all sites, corresponding to a refusal rate of <2%, indicating high acceptability of the protocol.

Across deployments, minute-resolution personal PM₂.₅, geolocation, and accelerometry data were successfully captured, yielding high-density spatiotemporal exposure datasets. Data completeness criteria (≥24 h of valid monitoring within the 120-hour deployment window) were satisfied in over 90% of deployments. Data loss events were infrequent and primarily attributable to battery depletion or isolated device malfunction under extreme environmental conditions, with no systematic differences observed across study sites. Ambient datasets achieved high completeness in The Gambia and Kenya (>85% data capture). Mozambique ambient capture was lower due to site-level connectivity constraints; however, application of predefined quality-control criteria requiring ≥75% completeness ensured that only monitoring periods meeting minimum data quality requirements were retained for analysis.

## Ambient monitoring and contextual exposure surfaces

Continuous ambient PM₂.₅ monitoring was successfully implemented at six health facilities, generating 15-minute resolution time series aligned with personal monitoring periods. These data provided stable background concentration profiles against which individual-level exposures were contextualised.

Ambient concentrations exhibited consistent seasonal variability across sites, reflecting underlying meteorological conditions and local emission dynamics. In Mozambique, comparison with co-located reference-grade BAM-1022 measurements demonstrated broadly concordant temporal patterns, providing contextual support for the ability of Clarity Node-S monitors to capture temporal variability in background PM₂.₅ concentrations, consistent with previous evaluations of these monitoring platforms (40). Ambient–personal concordance analyses demonstrated moderate associations across sites, with log–log regression slopes ranging from 0.12 to 1.06 and adjusted R^2^ values ranging from <0.01 to 0.56. Concordance was generally stronger under wet-season conditions, consistent with more homogeneous atmospheric mixing, while dry-season relationships were weaker and more variable. Across settings, slopes were frequently below unity, indicating that ambient monitoring captures background trends but incompletely represents individual-level variability. Detailed evaluation of these relationships has been reported elsewhere using the same dataset (41). These findings confirm that while fixed-site monitoring captures broad temporal trends, it incompletely represents individual-level exposure variability, supporting the need for person-resolved monitoring in heterogeneous environments.

## Calibration performance and cross-site harmonisation

Pre-deployment calibration against reference instrumentation demonstrated stable sensor performance, with Deming regression yielding mean scaling factors of 1.24 (SD 0.13), intercepts of −1.70 (SD 0.24), and coefficients of determination exceeding 0.72.

In-country co-location further improved inter-sensor agreement, with post-harmonisation scaling factors approaching unity (mean 1.03, SD 0.05) and R^2^ values exceeding 0.75 across sites. Devices exhibiting excessive drift or inconsistent performance during these procedures were excluded prior to deployment.

Application of calibration coefficients to minute-level data resulted in harmonised exposure estimates across devices and study settings, reducing inter-device variability while preserving sensitivity to short-duration exposure fluctuations.

## Microenvironment classification and mobility-linked exposure

Geolocation-informed classification enabled partitioning of exposure into home and away microenvironments for over 90% of deployments. Residential buffers were successfully defined across diverse settlement contexts, supporting consistent classification across sites.

Mobility patterns varied by setting, with rural participants spending a greater proportion of time within home geofences, while urban participants exhibited increased time in away environments associated with market activity, transport corridors, and informal employment. Accelerometer-derived motion data supported differentiation between stationary and active periods, enhancing contextual interpretation.

Microenvironmental partitioning suggested reproducible diurnal structure in exposure profiles across settings, with elevated concentrations observed during periods associated with domestic activity and mobility. These patterns highlight the contribution of behavioural and spatial dynamics to exposure variability beyond ambient background concentrations.

## Protocol acceptability and field feasibility

Participant adherence to the wear protocol was high across all study sites. The high levels of adherence observed in this study reinforce the value of incorporating community engagement and participant sensitisation as core components of exposure monitoring protocols. Observations recorded by field teams during deployment indicated that culturally adapted and discreet carrying designs contributed positively to participant acceptability and sustained compliance. These observations were supported by consistently high completion rates, low device refusal, and the successful integration of monitors into routine daily activities across study settings. A summary of implementation observations recorded by field teams is provided in Supplementary Table S1.Operational challenges included perceived heat burden during high-temperature periods and community curiosity regarding monitoring devices unfamiliar to participants. These were effectively mitigated through community sensitisation and routine fieldworker engagement. Device refusal was rare (*n* = 5), and no systematic differences in adherence were observed across countries.

Field implementation was further shaped by insights from fieldworkers, which provided critical context on the practical realities of conducting high-resolution air pollution monitoring in diverse sub-Saharan African settings. Initial participant concerns—particularly around the perceived presence of recording devices within sensor units—highlighted the importance of early and sustained community sensitisation. With targeted engagement and clear communication, these concerns were rapidly mitigated, and participants demonstrated high levels of acceptance, often integrating the wearable monitors into daily routines across domestic, occupational, and community environments. Field teams reported that participants carried devices during routine activities including market trading, agricultural work, and social gatherings, indicating successful embedding of the protocol within lived contexts. At the same time, operational challenges such as heat burden during peak dry-season conditions and social visibility of unfamiliar devices required adaptive field strategies, including mid-deployment check-ins and community-level reassurance. Notably, participants expressed sustained interest in their own exposure data, reflecting a broader demand for locally relevant environmental health information. These observations underscore that successful deployment of personal monitoring technologies in low-resource settings depends not only on technical robustness but also on trust-building, cultural alignment, and continuous engagement between research teams and communities.

## Data governance and secure implementation

All data were managed within a secure, harmonised framework across study sites. Encrypted data transfer protocols functioned as intended, with zero reported data breaches or losses. Geolocation data were stored separately from clinical records and linked through anonymised identifiers, ensuring compliance with GDPR-aligned governance requirements.

These procedures demonstrate the feasibility of implementing geolocation-enabled exposure monitoring within multi-country cohort studies while maintaining robust data protection standards.

## Synthesis

Together, these results demonstrate that the protocol generates high-resolution, behaviourally contextualised exposure data with strong cross-site comparability and robust data integrity, providing a reliable foundation for subsequent exposure–response analyses within cohort-based studies. These metrics collectively indicate high protocol fidelity across measurement, calibration, and data integration domains.

## Discussion

This study demonstrates that a climate-structured, personal–ambient air pollution monitoring protocol can be implemented with high data completeness, strong participant adherence, and robust cross-site comparability across three sub-Saharan African settings. The successful capture of minute-resolution personal PM₂.₅ data in over 90% of deployments, coupled with a refusal rate below 2%, indicates that intensive personal monitoring is operationally feasible when supported by culturally responsive deployment strategies and structured field protocols. These findings are consistent with emerging evidence demonstrating the feasibility of personal air pollution monitoring in low- and middle-income settings when supported by community engagement and appropriate deployment strategies (42, 43).

A central methodological insight arising from these results is the limited capacity of ambient monitoring to represent individual-level exposure variability. Moderate ambient–personal concordance, with slopes frequently below unity and substantial variability in explained variance (R^2^ < 0.01–0.56), indicates that fixed-site measurements capture broad temporal trends but systematically underestimate peak personal exposures. This pattern aligns with prior work showing that fixed-site monitors often fail to capture intra-urban and microenvironmental variability in exposure (44–46). This divergence was more pronounced under dry-season conditions, consistent with greater spatial heterogeneity and localised emission sources. Similar seasonal amplification of exposure heterogeneity has been reported in settings influenced by biomass combustion and dust resuspension (47, 48). These findings reinforce the need for person-resolved monitoring in settings where exposure is shaped by microenvironmental and behavioural factors and support the integration of personal monitoring within cohort-based exposure assessment frameworks.

The calibration and harmonisation framework demonstrated stable sensor performance across laboratory and field conditions. Pre-deployment calibration produced consistent scaling factors and strong agreement with reference instrumentation, while in-country co-location further reduced inter-device variability, yielding near-unity scaling factors and improved concordance. These results indicate that low-cost wearable platforms, when supported by structured calibration and quality assurance procedures, can generate internally consistent datasets suitable for epidemiological analysis. This is consistent with a growing body of literature demonstrating that low-cost sensors can achieve acceptable performance when supported by rigorous calibration and quality control frameworks (49, 50). Importantly, the observed stability across climatically distinct settings suggests that calibration architecture, rather than device class alone, is a primary determinant of measurement reliability.

Microenvironment classification achieved for most deployments enabled partitioning of exposure into home and mobility-linked domains, revealing context-specific differences in time–activity patterns. Rural participants spent a greater proportion of time within home environments, while urban participants exhibited increased mobility-associated exposure. The presence of reproducible diurnal structuring across sites further suggests that exposure variability is systematically patterned by daily routines rather than random fluctuation. These findings align with studies demonstrating that time–activity patterns and microenvironmental transitions are major determinants of personal exposure variability (38). The findings highlight the importance of integrating spatial and behavioural context into exposure assessment and demonstrate the added value of combining geolocation and accelerometry data with personal monitoring.

The implementation of ambient monitoring alongside personal measurements provided essential contextualisation of background concentrations and seasonal trends. While ambient data alone did not capture individual exposure variability, their integration enabled interpretation of temporal patterns and supported evaluation of measurement concordance. Similar hybrid monitoring approaches have been recommended to improve exposure characterisation in epidemiological studies (51, 52). This dual-scale monitoring approach strengthens the interpretability of exposure datasets and provides a foundation for future analyses examining the interaction between background concentrations and localised exposure drivers.

High levels of protocol adherence and minimal refusal rates underscore the importance of community engagement and participatory design in enabling intensive monitoring in diverse settings. The successful use of culturally adapted, discreet carrying designs, combined with community sensitisation and ongoing fieldworker engagement, mitigated potential barriers related to device visibility, heat burden, and unfamiliarity. These findings demonstrate that technical feasibility is closely linked to sociocultural acceptability, particularly in contexts where trust and community dynamics influence participation. This is consistent with evidence highlighting the importance of community engagement and culturally appropriate study design in environmental health research (53–55). Complementary qualitative work undertaken within the Kenya sites will provide further insight into participant and fieldworker experiences of wearable monitoring and the sociocultural dimensions of implementation.

Several limitations should be considered when interpreting these findings. Continuous co-location with reference-grade instrumentation was not feasible across all sites, limiting assessment of long-term sensor drift under sustained tropical conditions. Although calibration procedures reduced inter-device variability, residual measurement uncertainty may persist, particularly under high humidity or dust loading. The ≥24-hour data completeness threshold represents a pragmatic inclusion criterion; while most participants exceeded this duration, shorter monitoring periods may increase within-person variability. Future implementation at larger geographic scales would likely require streamlined calibration procedures, automated data transfer systems, and integration with existing health surveillance infrastructure to reduce operational costs and logistical burden. Finally, purposive sampling enhanced structural diversity but limits direct extrapolation to the full cohort without subsequent modelling.

The AE22 aethalometer used during laboratory calibration measures black carbon rather than total PM₂.₅ mass. Consequently, calibration procedures were intended primarily to improve inter-device consistency and temporal comparability rather than establish direct equivalence with gravimetric PM₂.₅ measurements. While black carbon is strongly associated with combustion-derived particulate pollution, particularly in biomass-burning environments, the two metrics are not interchangeable.

Despite these limitations, the protocol establishes a reproducible and adaptable framework for high-resolution exposure assessment in under-monitored environments. While implementation required investment in sensor calibration, fieldworker training, data management infrastructure, and community engagement, the methodological architecture is transferable across diverse settings and can be adapted to local logistical, technological, and resource constraints. The framework therefore provides a practical foundation for future exposure assessment initiatives in regions where conventional monitoring infrastructure remains limited. This contributes to ongoing efforts to expand high-resolution exposure assessment in regions where monitoring infrastructure remains limited (56). The combination of multi-season deployment, calibration harmonisation, geolocation-informed classification, and secure data governance enabled the generation of high-quality, behaviourally contextualised exposure data across multiple settings. These results challenge assumptions that rigorous exposure measurement is infeasible in low-resource contexts and demonstrate that methodologically robust monitoring can be achieved through integrated technical and operational design.

The implications of this work extend beyond protocol implementation to improving the validity of exposure–response inference in heterogeneous environments. By generating exposure data that reflect both temporal dynamics and behavioural context, the framework provides a foundation for more precise exposure–response modelling and reduces reliance on proxy-based estimates. This is particularly important given evidence that exposure misclassification can substantially bias exposure–response relationships (57, 58). This is particularly relevant in settings characterised by heterogeneous emission sources and limited monitoring infrastructure, where conventional approaches may obscure critical exposure gradients.

In summary, the PRECISE Air Quality protocol demonstrates that climate-structured, person-centred exposure monitoring can be implemented with high fidelity across diverse settings. The protocol generates data that are temporally resolved, spatially contextualised, and internally consistent, thereby enabling exposure–response inference with substantially reduced misclassification in environmental health research. By integrating calibration rigour, microenvironmental classification, and community engagement within a unified framework, this approach advances the methodological foundations required for precise and contextually grounded exposure–response inference in under-monitored regions.

## Conclusion

The PRECISE Air Quality protocol demonstrates that personal exposure monitoring can be embedded within maternal cohort infrastructures across diverse sub-Saharan African settings with high data fidelity and operational consistency. By integrating calibration, inter-sensor harmonisation, microenvironment classification, meteorological contextualisation, and culturally responsive implementation, the framework generated temporally resolved personal and ambient PM₂.₅ measurements suitable for robust epidemiological analysis.

Beyond feasibility, the protocol establishes a replicable methodological architecture for reducing exposure misclassification, capturing variability across contrasting seasonal climatic regimes, and linking environmental measurement to structural determinants central to women's health. In settings characterised by limited monitoring infrastructure and increasing climatic variability, person-centred exposure assessment is essential for strengthening the internal validity of environmental epidemiology.

By aligning technical rigour with community engagement and data governance safeguards, the PRECISE protocol advances equitable exposure science and supports the integration of exposure assessment frameworks that explicitly account for seasonal climatic variability within global maternal and newborn health research. Continued refinement, adaptation, and evaluation of such approaches will be essential to inform context-specific interventions and policy.

## Data Availability

The PRECISE and PRECISE-DYAD data are de-identified participant-level data. As permitted by existing data-sharing and collaboration agreements, data are available to academically active entities, including universities, non-governmental organisations and multilateral organisations, within the limits of the informed consent obtained. External and PRECISE network researchers may request access to data and samples, with requests reviewed through the PRECISE Data and Samples Access Committee. Requests for access should be directed to the PRECISE network at HYPERLINK “mailto:precise@kcl.ac.uk” precise@kcl.ac.uk and will be considered in accordance with the established data-access procedures.
