# Supplementary material for: An integrated climate-structured personal and ambient air pollution monitoring framework embedded within a pregnancy and postpartum cohort in Sub-Saharan Africa
Source: Front Glob Womens Health. 2026 Oct 5;7:1849628. doi: 10.3389/fgwh.2026.1849628 (PMC13679543; doi:10.3389/fgwh.2026.1849628)
Supplement: Supplementary file 1 [file Supplementaryfile1.docx]

**Supplementary Material**

**Supplementary Methods: PRECISE Air Quality Protocol**

**S1. Device Architecture and Sensor Configuration**

Personal exposure monitoring was conducted using wearable environmental monitoring devices integrating multiple sensing components within a single platform. Each device incorporated a Sensirion SPS30 optical particle counter capable of measuring particulate matter concentrations across PM₁, PM₂.₅, and PM₁₀ size fractions using laser scattering technology. Additional sensors included modules for temperature and relative humidity, a global positioning system (GPS) receiver for geolocation tracking, and a tri-axial accelerometer for motion detection.

Devices were configured to record data at one-minute resolution, balancing temporal granularity with battery capacity sufficient for continuous operation over five-day monitoring periods. Internal data logging allowed uninterrupted acquisition during deployment, with data subsequently downloaded following device retrieval.

To ensure suitability for field conditions, devices were enclosed in protective housings designed to minimise exposure to dust, humidity, and mechanical shock while maintaining adequate airflow to the particle inlet. This configuration supported stable operation across diverse climatic environments encountered in The Gambia, Kenya, and Mozambique.

**S2. Wear Protocol and Field Implementation**

Wearable monitors were housed in bespoke cross-body shoulder bags developed through a participatory co-design process involving local research teams and study participants. The design prioritised comfort, discretion, and cultural acceptability, incorporating lightweight materials and adjustable straps to facilitate continuous wear.

Participants were instructed to carry the monitoring device during waking hours for five consecutive days per deployment period. During sleep, devices were positioned approximately one metre from the participant’s sleeping location to approximate breathing-zone exposure while ensuring comfort and safety.

Fieldworkers provided standardised verbal instructions and demonstrated correct positioning of devices at the time of deployment. Mid-deployment checks were conducted to verify adherence to the protocol and to identify any technical issues requiring intervention. This approach supported high levels of compliance across study settings.

**S3. Calibration and Sensor Harmonisation Framework**

Calibration of personal monitoring devices was conducted using a two-stage framework comprising reference-site calibration and in-country inter-sensor harmonisation.

Pre-deployment calibration was performed through repeated co-location of devices with a reference aethalometer (AE22) at the Marylebone Road monitoring station in London. Calibration coefficients were derived using Deming regression, which accounts for measurement error in both reference and sensor observations.

Let $Y_{i}$denote the reference PM₂.₅ concentration and $X_{i}$the corresponding sensor measurement. The calibration model is defined as:

$$\boldsymbol{Y}_{\boldsymbol{i}}\boldsymbol{=\alpha+\beta}\boldsymbol{X}_{\boldsymbol{i}}\boldsymbol{+}\boldsymbol{\epsilon}_{\boldsymbol{i}}$$

where $\boldsymbol{\epsilon}_{\boldsymbol{i}}$represents normally distributed error. The variance ratio is defined as $\boldsymbol{\lambda=}\boldsymbol{\sigma}_{\boldsymbol{X}}^{\boldsymbol{2}}\mathbf{/}\boldsymbol{\sigma}_{\boldsymbol{Y}}^{\boldsymbol{2}}$**.**

The Deming estimator for the slope parameter is given by:

$$\hat{\boldsymbol{\beta}}\boldsymbol{=}\frac{\boldsymbol{S}_{\boldsymbol{YY}}\boldsymbol{-\lambda}\boldsymbol{S}_{\boldsymbol{XX}}\boldsymbol{+}\sqrt{\left( \boldsymbol{S}_{\boldsymbol{YY}}\boldsymbol{-\lambda}\boldsymbol{S}_{\boldsymbol{XX}}\boldsymbol{)}^{\boldsymbol{2}}\boldsymbol{+4}\boldsymbol{\lambda}\boldsymbol{S}_{\boldsymbol{XY}}^{\boldsymbol{2}} \right.}}{\boldsymbol{2}\boldsymbol{S}_{\boldsymbol{XY}}}$$

where $\boldsymbol{S}_{\boldsymbol{XX}}$**,** $\boldsymbol{S}_{\boldsymbol{YY}}$**,** and $\boldsymbol{S}_{\boldsymbol{XY}}$denote sample covariances**.**

Following laboratory calibration, devices were co-located for five days at each study site prior to deployment to evaluate inter-sensor agreement under local environmental conditions. Devices exceeding predefined drift thresholds were excluded from deployment.

Cross-site harmonisation was achieved through implementation of identical deployment, calibration, and processing procedures across all participating countries. Sensor firmware versions, logging intervals, calibration schedules, and quality assurance criteria were standardised prior to deployment. Following download, all datasets were processed centrally using a harmonised analytical pipeline that applied uniform calibration coefficients, completeness criteria, exposure metric definitions, and geofencing algorithms. This approach minimised methodological variability between sites and ensured that observed differences reflected underlying exposure patterns rather than analytical inconsistencies.

This calibration framework was implemented to minimise systematic bias and ensure comparability of measurements across devices and study settings.

**S4. Data Processing and Quality Control Procedures**

All monitoring data were processed using a harmonised analytical pipeline applied consistently across study sites. Devices were synchronised to local time prior to deployment, and timestamp verification was conducted during data processing to ensure alignment between personal monitoring, ambient measurements, and meteorological variables.

Data completeness criteria required a minimum of twenty-four hours of valid PM₂.₅ observations within each five-day monitoring period. For ambient monitoring data, a separate completeness criterion required ≥75% of expected observations within the monitoring period before inclusion in analysis. Records not meeting this threshold were excluded from analysis.

Extreme values were Winsorised at the first and ninety-ninth percentiles to reduce the influence of implausible artefacts while preserving high-intensity exposure events. Quality control procedures also included validation of sensor operating ranges, inspection of anomalous readings, and verification of calibration coefficients prior to analysis.

**S5. Microenvironment Classification and Geolocation Processing**

Geolocation data collected through GPS modules were processed to classify exposure into domestic and mobility-linked microenvironments. Participant residences were geocoded during field visits, and a circular buffer with a radius of fifty metres centred on the residence was used to define the home microenvironment.

All GPS coordinates falling within this buffer were classified as home locations, while coordinates outside the buffer were classified as away environments representing mobility-related exposures, including occupational and community activities.

The fifty-metre buffer radius was selected to accommodate typical GPS positional uncertainty under field conditions and variation in residential compound size across rural and peri-urban settings. Accelerometer-derived motion data were used to support contextual interpretation by distinguishing between stationary and active periods.

**S6. Community Engagement and Field Adaptation**

Community sensitisation activities were conducted prior to field deployment to support participant understanding and acceptance of monitoring procedures. Engagement included meetings with local leaders, explanation of study objectives, and demonstration of monitoring devices.

Field teams maintained regular contact with participants throughout deployment periods to address questions, reinforce protocol adherence, and resolve technical issues. This approach facilitated high compliance and minimised device loss or misuse.

Feedback from participants and fieldworkers indicated that the use of culturally adapted device carriers and clear communication strategies contributed to acceptability across diverse study settings.

**S7. Summary of Field Implementation Observations**

| **Theme** | **Observation** |
| --- | --- |
| Device acceptability | Most participants rapidly adapted to carrying monitors |
| Community perceptions | Initial concerns regarding recording functions resolved through sensitisation |
| Comfort | Some reports of heat burden during peak dry-season conditions |
| Agricultural activities | Occasional logistical challenges during field work |
| Adherence | High completion rates observed across all countries |
| Participant interest | Participants frequently requested information about their exposure measurements |

Supplementary Table S1: Fieldworker Implementation Observations
