# Supplementary material for: An integrated climate-structured personal and ambient air pollution monitoring framework embedded within a pregnancy and postpartum cohort in Sub-Saharan Africa
Source: Front Glob Womens Health. 2026 Oct 5;7:1849628. doi: 10.3389/fgwh.2026.1849628 (PMC13679543; doi:10.3389/fgwh.2026.1849628)
Supplement: Supplementary file 2 [file Supplementaryfile2.docx]

**Supplementary Material – PRECISE & PRECISE DYAD Network Members**

**S8. The PRECISE Network**

| **Institution** | **PRECISE Network members** |
| --- | --- |
| **In-country teams** | |
| THE GAMBIA: Medical Research Council Unit The Gambia at the London School of Hygiene and Tropical Medicine, Fajara | Umberto D’Alessandro; Anna Roca; Hawanatu Jah; Andrew Prentice; Melisa Martinez-Alvarez; Brahima Diallo; Abdul Sesay; Sambou Suso; Baboucarr Njie; Fatima Touray; Yahaya Idris; Fatoumata Kongira; Modou F.S. Ndure; Lawrence Gibba; Abdoulie Bah; Yorro Bah |
| KENYA: Aga Khan University, Nairobi | Marleen Temmerman; Angela Koech; Patricia Okiro; Geoffrey Omuse; Grace Mwashigadi; Consolata Juma; Joseph Mutunga; Moses Mukhanya; Onesmus Wanje; Isaac Mwaniki; Marvin Ochieng; Emily Mwadime; Alice Kombo; Nathan Barreh; Christine Dama Baya; Juma Gumbo; Grace Maitha; Irene Chadi; Sharon Konde |
| MOZAMBIQUE: Centro de Investigação em Saúde de Manhiça, Manhiça | Esperança Sevene; Corssino Tchavana; Salesio Macuacua; Anifa Vala; Helena Boene; Lazaro Quimice; Sonia Maculuve; Inacio Mandomando |
| **Central co-ordinating team** | |
| Department of Women and Children’s Health, School of Life Course Sciences, Faculty of Life Sciences and Medicine, King’s College London | Peter von Dadelszen; Laura A. Magee; Rachel Craik; Marie-Laure Volvert; Hiten Mistry; Thomas Mendy |
| Donna Russell Consulting | Donna Russell |
| **Co-Investigator team** | |
| Midlands State University, Zimbabwe | Prestige Tatenda Makanga; Liberty Makacha; Reason Mlambo |
| King’s College London | Lucilla Poston; Rachel Tribe; Sophie Moore; Tatiana Salisbury |
| University of Oxford | Aris Papageorghiou; Alison Noble; Rachel Craik |
| London School of Hygiene and Tropical Medicine | Hannah Blencowe; Veronique Filippi; Joy Lawn; Matt Silver; Joseph Akuze; Ursula Gazeley |
| St George’s, University of London | Judith Cartwright; Guy Whitley; Sanjeev Krishna |
| University of British Columbia | Marianne Vidler; Jing (Larry) Li; Jeff Bone; Mai-Lei (Maggie) Woo Kinshella; Domena Tu; Ash Sandhu; Kelly Pickerill |
| Eduardo Mondlane University, Maputo | Carla Carillho |
| Imperial College London | Benjamin Barratt |

**S9. Membership of the PRECISE-DYAD Network**

| **Institution** | **PRECISE-DYAD Network members** |
| --- | --- |
| King's College London, UK | Peter von Dadelszen; Laura A. Magee; Rachel Craik; Hiten Mistry; Marie-Laure Volvert; Lucilla Poston; Rachel Tribe; Sophie Moore; Tatiana Salisbury |
| Aga Khan University, Kenya | Patricia Okiro; Geoffrey Omuse; Moses Mukhanya; Patrick Kulundu; Irene Chadi; Nathan Barreh; Ali Thoya; Michaelina Changawa; Juma Gumbo; Alice Kombo; Sarah Maitha; Olivia Kasuu; Joy Jefwa; Salim Omar; Isaac Menza; Belinder Orero; Grace Wairimu |
| Medical Research Council Unit The Gambia at the London School of Hygiene and Tropical Medicine, The Gambia | Umberto D'Alessandro; Anna Roca; Hawanatu Jah; Andrew Prentice; Melisa Martinez-Alvarez; Brahima Diallo; Abdul Sesey; Sambou Suso; Fatima Touray; Modou F.S. Ndure; Yahaya Idris; Baboucarr Njie; Fatoumata Kongira; Lawrence Gibba; Abdoulie Bah; Yorro Bah |
| Centro de Investigação em Saúde de Manhiça, Mozambique | Esperança Sevene; Corssino Tchavana; Salesio Macuacua; Jovito Nunes; Valdemiro Escola; Charfudin Sacoor |
| Midlands State University, Zimbabwe | Prestige Tatenda Makanga; Liberty Makacha |
| London School of Hygiene and Tropical Medicine, UK | Hannah Blencowe; Veronique Filippi; Joy Lawn; Jaya Chandna |
| St George's University of London, UK | Asma Khalil |
| University of British Columbia, Canada | Marianne Vidler; Jing (Larry) Li; Jeff Bone; Mai-Lei (Maggie) Woo Kinshella; Domena Tu; Akshdeep Sandhu; Kelly Pickerill |
| Imperial College London, UK | Ben Barratt |
| University of Liverpool/Liverpool School of Tropical Medicine, UK | Melissa Gladstone; Dorcas Magai |
| ISGlobal, Spain | Cathryn Tonne; Ariadna Curto; Ariadna Moreno |
| C-Squared Development | Chris Clarke |
